# Analysis of the Indole Diterpene Gene Cluster for Biosynthesis of the Epoxy-Janthitrems in *Epichloë* Endophytes

**DOI:** 10.3390/microorganisms7110560

**Published:** 2019-11-13

**Authors:** Emma J. Ludlow, Simone Vassiliadis, Piyumi N. Ekanayake, Inoka K. Hettiarachchige, Priyanka Reddy, Tim I. Sawbridge, Simone J. Rochfort, German C. Spangenberg, Kathryn M. Guthridge

**Affiliations:** 1 Agriculture Victoria Research, AgriBio, Centre for AgriBioscience, Bundoora, Victoria 3083, Australia; emma.ludlow@agriculture.vic.gov.au (E.J.L.); simone.vassiliadis@agriculture.vic.gov.au (S.V.); piyumi.ekanayake@agriculture.vic.gov.au (P.N.E.); inoka.hettiarachchige@agriculture.vic.gov.au (I.K.H.); priyanka.reddy@agriculture.vic.gov.au (P.R.); tim.sawbridge@agriculture.vic.gov.au (T.I.S.); simone.rochfort@agriculture.vic.gov.au (S.J.R.); german.spangenberg@agriculture.vic.gov.au (G.C.S.); 2 School of Applied Systems Biology, La Trobe University, Bundoora, Victoria 3083, Australia

**Keywords:** fungi, *Epichloë*, endophyte, mycotoxin, indole diterpene, epoxy-janthitrems

## Abstract

Epoxy-janthitrems are a class of indole diterpenes with structural similarity to lolitrem B. Two taxa of asexual *Epichloë* endophytes have been reported to produce epoxy-janthitrems, *Lp*TG-3 (*Lolium perenne* Taxonomic Group 3; e.g., NEA12) and *Lp*TG-4 (e.g., E1). *Epichloë* epoxy-janthitrems are not well understood, the biosynthetic pathway and associated gene complement have not been described and while the literature suggests they are associated with superior protection against pasture insect pests and are tremorgenic in grazing mammals, these properties have not been confirmed using isolated and purified compounds. Whole genome sequence analysis was used to identify candidate genes for epoxy-janthitrem biosynthesis that are unique to epoxy-janthitrem producing strains of *Epichloë*. A gene, *jtmD*, was identified with homology to aromatic prenyl transferases involved in synthesis of indole diterpenes. The location of the epoxy-janthitrem biosynthesis gene cluster (JTM locus) was determined in the assembled nuclear genomes of NEA12 and E1. The JTM locus contains cluster 1 and cluster 2 of the lolitrem B biosynthesis gene cluster (LTM locus), as well as four genes *jtmD, jtmO, jtm01*, and *jtm02* that are unique to *Epichloë* spp. that produce epoxy-janthitrems. Expression of each of the genes identified was confirmed using transcriptome analysis of perennial ryegrass-NEA12 and perennial ryegrass-E1 symbiota. Sequence analysis confirmed the genes are functionally similar to those involved in biosynthesis of related indole diterpene compounds. RNAi silencing of *jtmD* and in planta assessment in host-endophyte associations confirms the role of *jtmD* in epoxy-janthitrem production. Using LCMS/MS technologies, a biosynthetic pathway for the production of epoxy-janthitrems I–IV in *Epichloë* endophytes is proposed.

## 1. Introduction

Perennial ryegrass (*Lolium perenne* L.) is often infected with fungal endophytes that include sexual *Epichloë* species and their asexual derivatives. In the case of asexual *Epichloë* spp. endophytes, the host grass–endophyte interaction is considered mutualistic as both the plant and the fungus receive benefits [1]. The plant provides the fungus with a means for survival and reproduction and the fungus protects this environment by producing compounds that aid the defence of the host plant. While the *Lolium*–*Epichloë* interaction produces compounds beneficial for plant growth and persistence, in a pastoral agriculture scenario, this interaction can produce compounds detrimental to animal health and production. These detrimental compounds are responsible for conditions such as tall fescue toxicosis, caused by ingestion of tall fescue infected with ergovaline-producing strains of *Epichloë coenophiala*; and ryegrass staggers, caused by indole diterpene producing strains of *Epichloë festucae* var. *lolii* (*Lolium perenne* taxonomic group 1, *Lp*TG-1), and *Lp*TG-3 [2,3].

Perennial ryegrass forms natural associations with asexual fungal endophytes from *Lp*TG-1*, Lp*TG-2 [4], also referred to as *Epichloë hybrida* [5], and *Lp*TG-3 [6]. The association between perennial ryegrass and *Lp*TG-1 is one of the most commonly studied grass-endophyte interactions because of its importance in agricultural pastoral systems in Australia, New Zealand, and the USA. Consequently, the search for endophyte strains that do not produce compounds detrimental to animal health has led to the discovery and characterisation of novel endophyte strains [6,7,8].

**Figure 1:**
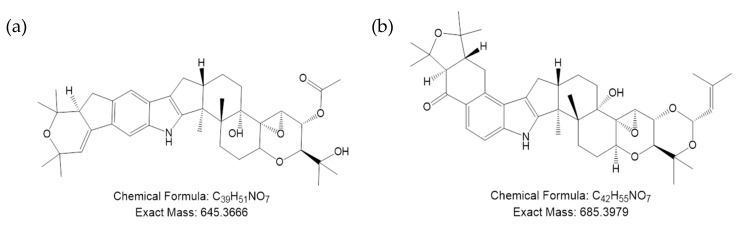
Structure, chemical formula and exact mass of (**a**) 11,12-epoxy-janthitrem G (epoxy-janthitrem I) and (**b**) lolitrem B.

*Lp*TG-3 strains such as NEA12 and AR37 (also known as Lp14) [4,9,10], do not produce lolitrem B, ergovaline or peramine, but do produce a group of indole diterpenes with structural similarity to lolitrem B called epoxy-janthitrems (Figure 1) [11]. In the literature to date, two taxa of asexual *Epichloë* endophytes have been reported to produce epoxy-janthitrems, *Lp*TG-3 and *Lp*TG-4 [6,7]. Although not as potent as lolitrem B, epoxy-janthitrems are the most likely cause of ryegrass staggers in livestock-grazing AR37 endophyte-infected pasture [12,13,14]. They may also be the compound responsible for the insect control properties exhibited by *Lp*TG-3 endophyte-infected ryegrass pastures [15,16,17]. This bioprotective property can be exploited in pastoral agriculture as part of an integrated pest management system [18].

Janthitrems were first isolated from *Penicillium janthinellum* strains obtained from pastures in which ryegrass staggers had been observed [19,20]. *Penicillium* species, which produce a number of tremorgenic mycotoxins, were thought to be the cause of ryegrass staggers until it was later proven to be *Epichloë*-derived lolitrem B [19,21]. The first evidence that janthitrems and lolitrem B share a common pathway came from an experiment in which *P. janthinellum* provided with C^14-^labelled paxilline showed incorporation of the radiolabel into janthitrem B, indicating that paxilline is a likely precursor for janthitrem B biosynthesis [22,23]. Schardl et al. [24] suggested that, for *Epichloë* endophytes, janthitrems are synthesised from terpendole I, which is also a precursor of lolitrem B biosynthesis. Tapper et al. [11] first identified the novel janthitrem named 11,12-epoxy-janthitrem G (epoxy-janthitrem I) from AR37 endophyte-infected perennial ryegrass. Epoxy-janthitrem I is the major janthitrem alkaloid produced by perennial ryegrass endophytes.

Indole diterpenes are a diverse group of secondary metabolites produced by filamentous fungi. The best characterised are from *Penicillium*, *Aspergillus*, *Claviceps* and *Epichloë* species; however, the number of species in which indole diterpenes have been identified continues to increase [25]. The indole diterpenoid compounds comprise a cyclic diterpene-derived skeleton (derived from four isoprene units, i.e., geranylgeranyl diphosphate (GGDP)) and an indole moiety (derived from tryptophan or a tryptophan precursor) [26,27]. The structural diversity of these metabolites is due to the different patterns of ring substitutions, different ring stereochemistry and additional prenylations [27]. In the context of their natural ecosystem, indole diterpenes act as bioprotectants, defending the fungus or protecting the host plant against herbivory by animals and insects. Many are potent mammalian toxins, and some have been shown to have antimicrobial and insecticidal properties [28]. Thus, production of indole diterpenes offers evolutionary advantages for the producing organism and its host plant.

Since the identification of the genes and biosynthetic pathway for paxilline in *P. paxilli* [29,30,31], nine gene clusters for the biosynthesis of the paspaline derived indole diterpenes have been identified, including those for aflatrems (*Aspergillus flavus* and *Aspergillus oryzae*) [32,33], lolitrems (*E. festucae*) [34,35], terpendoles (*Tolypocladium album*) [36], penitrems (*P. crustosum* and *P. simplicissimum*) [33,37], shearinines (*P. janthinellum*) [33] and paspalitrems (*Claviceps paspali*) [25]. All these gene clusters contain orthologues of the *paxG*, *paxM*, *paxC* and *paxB* genes whose enzyme products are collectively responsible for the biosynthesis of paspaline. The known biosynthetic gene clusters for the different paspaline-derived indole diterpene families show little synteny, however, each of the biosynthetic pathways share a common, conserved set of genes that encode enzymes for the first steps in indole diterpene biosynthesis. The pathways then diverge to create the diversity of indole diterpenes [25].

While some literature on the properties of epoxy-janthitrem-producing endophytes exists, janthitrems themselves are not well understood when compared to other alkaloid groups synthesised by *Epichloë* endophytes. For example, while the genes and biosynthetic pathway for lolitrem B [34,35,38] and ergovaline [39] have been described and extensively characterised, the epoxy-janthitrem biosynthetic pathway and associated gene complement have not been described.

The literature suggests epoxy-janthitrems are associated with superior protection against pasture insect pests and are tremorgenic to grazing mammals, however, these properties have not been confirmed using isolated and purified compounds. This is in part because they are too unstable to isolate and purify in large enough quantities to study [15]. If the genetic basis for epoxy-janthitrem production is understood, it then becomes possible to characterise their properties. In this study, we identify the JTM locus (epoxy-janthitrem biosynthetic gene cluster) and the genes required for epoxy-janthitrem biosynthesis in asexual *Epichloë* endophytes. We functionally characterise the gene *jtmD*, using RNAi-mediated gene knockdown followed by liquid chromatography-mass spectrometry (LC-MS) analysis of transgenic endophytes in planta, to confirm the role of *jtmD* in epoxy-janthitrem I production. Finally, we propose a parsimonious pathway for biosynthesis of epoxy-janthitrems by *Epichloë* endophytes.

## 2. Materials and Methods

### 2.1. Fungal Strains and Growth Conditions

The properties of *Epichloë* endophytes used in this study are shown in Table 1. Endophyte cultures were grown either on potato dextrose agar (PDA) (Sigma-Aldrich, St. Louis, MO, USA), or in potato dextrose broth (PDB) at 22 °C, 150 rpm in the dark for a period of 7–10 days depending on growth rate.

### 2.2. Identification of a Candidate Gene for Epoxy-Janthitrem Biosynthesis, *jtmD*, and the JTM Locus

#### 2.2.1. DNA extraction and short-read sequencing on Illumina HiSeq2000 sequencing platform

Genomic DNA was extracted from lyophilized endophyte mycelia using a cetyltrimethylammonium bromide (CTAB) based extraction method [40], and the quality and quantity of the DNA was assessed by agarose gel electrophoresis and specific absorbance measurements using NanoDrop 2000 Spectrophotometer (Thermo Fisher Scientific, Walthman, MA, USA). Genomic DNA was fragmented in a Covaris instrument (Woburn, MA, USA) to an average size of 100–900 bp. For each endophyte DNA sample, paired-end libraries with inserts c. 400 bp in size were prepared using the standard protocol (TruSeq DNA Sample Prep V2 Low Throughput; Illumina Inc., San Diego, CA, USA) with paired-end adaptors. Library quantification was performed using the KAPA library quantification kit (KAPA Biosystems, Wilmington, MA, USA) and sequenced using the HiSeq2000 platform (Illumina Inc., San Diego, CA, USA) following the standard manufacturer’s protocol.

#### 2.2.2. DNA Extraction and Long-Read Sequencing on PacBio and MinIon Sequencing Platforms

Genomic DNA was extracted from endophyte mycelia snap frozen in liquid N_2_ using a modified CTAB extraction method [41]. The quality and specific absorbance measurements of DNA was assessed as explained above. DNA Quantification was measured by Qubit (Thermo Fisher Scientific, Walthman, MA, USA) according to manufacturer’s instructions. Genomic libraries were prepared and sequenced according to 20 kb SMRTbell library preparation kit (Pacific Biosciences, Menlo Park, CA, USA) and ligation sequence preparation kit (SQK-LSK109; Oxford Nanopore, Oxford, UK) for PacBio and MinIon, respectively.

#### 2.2.3. Sequence Assembly and JTM Locus Identification

PacBio generated long-reads of NEA12 were filtered, assembled (assuming the genome size as 35 Mb), polished using SMRT Portal software HGAP assembler [42], and split into 500,000 bp pieces for read mapping. For MinIon generated reads of E1, sequence correction, trimming and assembly was performed using the long-read assembler Canu v.1.8 [43], assuming the genome size of 35 Mb. Scaffolded assembly produced by Canu was polished with genome assembly improvement and variant detection tool Pilon v. 1.23 [44], using short-reads generated from Illumina HiSeq 2000 sequencing platform. Final E1 genome assembly was split into 500,000 bp pieces for read mapping. Illumina generated sequence reads were quality trimmed using the Gydle nuclear program (Gydle Inc., Québec City, QC, Canada) such that all reads ≥50 bases long were retained using a base quality cut-off value of 20. All high-quality reads for a given endophyte strain were mapped to the NEA12 PacBio contig 3 (247,475 bp) using the Gydle ‘nuclear’ aligner version 3.2.1 (Gydle Inc., Québec City, QC, Canada). Reads were mapped with settings: l 50 (length of overlap); s 25 (sensitivity); k 13 (kmer length); m 6 (maximum number of mis-matches); F 3 (filter settings). Alignments were visualised with Gydle program vision version 2.6.14 (Gydle Inc., Québec City, QC, Canada).

The JTM locus was identified, first by identifying the location of the *jtmD* gene and then annotating the region using a combination of both Augustus [45], gene prediction and manual annotation using the known gene sequences of LTM genes [35,38], and *jtmD*. Each of the predicted genes were then characterised by BLASTp analysis and comparisons with genes from other indole diterpene gene clusters performed by multiple sequence alignment using MUSCLE v. 3.8.31 [46], with default parameters. To reconstruct tree topology ML analysis was used as implemented in MEGA X [47], using the maximum likelihood method and JTT matrix-based model [48], using the partial deletion option (all positions with less than 95% site coverage were eliminated). The tree with the highest log likelihood is shown. Nearest-neighbour-interchange (NNI) was performed as a heuristic method. The internal branch support was assessed by a search of 500 bootstrapped sets of data.

#### 2.2.4. Transcriptome Expression of Genes Located Within the JTM Locus

Endophyte devoid (E^−^) perennial ryegrass cultivar Alto seeds, harvested from endophyte devoid parent plants, and those containing the *Epichloë* endophyte strains SE and NEA12 were obtained from New Zealand Agriseeds (NZA), Christchurch, New Zealand. Seeds were prepared, RNA extracted and RNA libraries constructed as described [49]. Four biological replicates were prepared for each endophyte strain. The Impact_04_-E1 transcriptome is described by Shinozuka et al. [50]. Raw sequence reads were trimmed using the Gydle ‘nuclear’ program and all high-quality reads were mapped to E1 JTM locus as detailed above (Gydle Inc., Québec City, QC Canada).

### 2.3. *jtmD* Knockdown Constructs and Transformation

#### 2.3.1. Fungal and Bacterial Strains and Culture Conditions

*Epichloë* endophyte strain E1, was the endophyte strain of choice due to the toxin profile (Table 1) and fast in vitro growth rate, for *jtmD* knockdown. *Escherichia coli* strain, DH5α, was used for construction and maintenance of plasmid constructs (Thermo Fisher Scientific, Walthman, MA, USA) and were grown at 37 °C on Luria-Bertani (LB) agar (5 g yeast extract, 10 g tryptone, 10 g NaCl in 1 L of ddH_2_O) plates or broth. PDA and LB media were supplemented with appropriate antibiotics (50 μg kanamycin, 50 μg/mL spectinomycin).

#### 2.3.2. Plasmid Construction

The plasmid, pDONR 221 and gene cassettes were supplied/synthesised by Invitrogen (Thermo Fisher Scientific, Walthman, MA, USA). Gene cassettes contained inverted repeats of *jtmD* from E1 (*jtmD*_129 bp and *jtmD*_432 bp) separated by a 147 bp spacer (cutinase gene intron from *Magnaporthe grisea*), flanked by *att*B1 and *att*B2 recombination sites. The Gateway™-enabled destination vector (pEND0002) was supplied by Agriculture Victoria Research (Thermo Fisher Scientific, Walthman, MA, USA) [49]. To generate entry clones, gene cassettes (inverted repeats of candidate gene sequences, *jtmD*_129 bp and *jtmD*_432 bp, separated by the 147 bp spacer and containing *att*B1 and *att*B2 sites), were cloned into the pDONR 221 vector using BP clonase (Thermo Fisher Scientific, Waltham, MA, USA) according to manufacturer’s instructions. The final RNA silencing vectors were produced by LR clonase reaction, following manufacturer’s recommendations (Thermo Fisher Scientific, Walthman, MA, USA), between the entry vectors carrying the *jtmD* candidate gene sequences (*jtmD*_129 bp and *jtmD*_432 bp) and the Gateway™-enabled destination vector, pEND002. The structures of all expression clones were verified by restriction enzyme analysis and Sanger sequencing.

#### 2.3.3. Isolation of Fungal Protoplasts

Mycelia were harvested, under sterile conditions, by filtration through layers of miracloth lining a funnel and washed 3 times with 30 mL of sterile ddH_2_O. Mycelia were washed with 10 mL of OM buffer (1.2 M MgSO_4_.7H_2_O, 10 mM Na_2_HPO_4_, 100 mM NaH_2_PO_4_.2H_2_O, pH 5.8) and transferred to a sterile 250 mL plastic vessel. Freshly prepared 10 mg/mL Glucanex (30 mL) (Sigma-Aldrich, St. Louis, MO, USA) in OM was added and incubated for 18 h at 30 °C with gentle shaking (80 rpm). The glucanex/protoplast solution (30 μL) was examined under a microscope to confirm successful digestion. Protoplasts were filtered through miracloth in a funnel, into 15 mL sterile glass centrifuge tubes (Gentaur, Belgium), and placed on ice. Each tube was carefully overlayed with 2 mL of ST buffer (0.6 M sorbitol, 100 mM Tris-HCl, pH 8.0) and centrifuged (Avanti^®^ J-25I; Beckman Coulter, Brea, CA, USA) (5000 rpm for 5 min at 4 °C). Following centrifugation, protoplasts formed a white layer between the glucanex solution and ST buffer, and this layer was carefully removed. STC buffer (1 M sorbitol, 50 mM CaCl_2_·2H_2_O, 50 mM Tris-HCl, pH 8.0) (5 mL) was added to the protoplast solution in fresh sterile glass tubes. Samples were gently inverted once and centrifuged (5000 rpm for 5 min at 4 °C). Protoplast pellets were pooled with 5 mL of STC buffer and centrifugation was repeated (5000 rpm for 5 min at 4 °C) until only one pellet remained. Excess STC buffer was removed, and the final protoplast pellet was re-suspended in 500 μL of STC buffer. Protoplast concentration was estimated by diluting protoplasts (1/100 and/or 1/1000 in STC buffer) and counting using 40× magnification (Olympus, BX41) and Reichert Bright-Line hemocytometer with improved Neubauer rulings (Hausser Scientific, Horsham, PA, USA). Protoplasts were diluted with STC to 1.25 × 10^8^ protoplasts/mL.

#### 2.3.4. PEG-mediated Fungal Protoplast Transformation

High quality plasmid DNA of the RNA silencing vectors was generated using PureYield™ Plasmid Midiprep System (Promega, Madison, WI, USA), according to manufacturers’ instructions. Aliquots (80 μL) of diluted protoplasts (1.25 × 10^8^ protoplasts/mL), were prepared under sterile conditions, on ice. To each aliquot, 2 μL 50 mM spermidine (Sigma-Aldrich, St. Louis, MO, USA), 5 μL heparin at 5 mg/mL, prepared in STC buffer (Sigma-Aldrich, St. Louis, MO, USA), 10 μg plasmid DNA (1 μg/μL, not exceeding 20 μL) and 20 μL 70% (*w*/*v*) PEG solution (70% (*w*/*v*) PEG 4000, 10 mM Tris-HCl pH 8.0, 10 mM CaCl_2_), was added. Eppendorf tubes were gently mixed and incubated on ice for 30 min. Following the addition of 1.5 mL STC buffer, protoplasts were mixed and centrifuged (Eppendorf 5424 R; Eppendorf, Hamburg, Germany) (5000 rpm for 5 min at 4 °C). The supernatant was removed and protoplasts were resuspended in 500 μL regeneration medium II (RG II) (304 g/L sucrose, 1 g/L KH_2_PO_4_, 1 g/L NH_4_NO_3_, 1 g/L NaCl, 0.25 g/L anhydrous MgSO_4_, 0.13 g/L CaCl_2_.2H_2_O, 1 g/L yeast extract, 12 g/L PD, 1 g/L peptone, 1 g/L acid hydrolysate of casein) and incubated overnight (22 °C, dark, 45 rpm).

#### 2.3.5. Fungal Protoplast Regeneration

Overnight protoplast solution (200 μL) was incubated with 800 μL 40% (*w*/*v*) PEG solution (40% (*w*/*v*) PEG 4000, 1 M sorbitol, 50 mM Tris-HCl pH 8.0, 50 mM CaCl_2_), at room temperature for 15 min. Molten (50 °C) 0.4% RG II (5 mL) (304 g/L sucrose, 1 g/L KH_2_PO_4_, 1 g/L NH_4_NO_3_, 1 g/L NaCl, 0.25 g/L anhydrous MgSO_4_, 0.13 g/L CaCl_2_.2H_2_O, 1 g/L yeast extract, 12 g/L PD, 1 g/L peptone, 1 g/L acid hydrolysate of casein, 4 g/L agarose) containing 100 of the protoplast/PEG mixture was spread evenly across 0.6% RG II agarose petri dishes (304 g/L sucrose, 1 g/L KH_2_PO_4_, 1 g/L NH_4_NO_3_, 1 g/L NaCl, 0.25 g/L anhydrous MgSO_4_, 0.13 g/L CaCl_2_.2H_2_O, 1 g/L yeast extract, 12 g/L PD, 1 g/L peptone, 1 g/L acid hydrolysate of casein, 6 g/L agarose) containing 100 μg/mL hygromycin B. Representative RG II petri dishes without hygromycin B assessed endophyte viability. All Petri dishes were incubated at 22 °C in the dark for 4–6 weeks until regeneration was observed.

#### 2.3.6. Molecular Analysis of Transformed Endophytes

Individual regenerated colonies were transferred onto petri dishes containing PDA with 200 μg/mL hygromycin B selection and incubated (22 °C, dark, 10–12 days). Hygromycin resistant colonies were grown in 250 mL sterile culture vessels with 50 mL PD broth and 100 μg/mL hygromycin B (22 °C, dark, 150 rpm, 10–12 days) and mycelia were harvested, under sterile conditions, by filtration through layers of miracloth lining a funnel and washed with 30 mL of sterile M9 phosphate buffer (1 g/L NH_4_Cl, 11 g/L Na_2_HPO_4_.7H_2_O, 3 g/l KH_2_PO_4_, 5 g/L NaCl). Washed mycelia were transferred to 15 mL sterile conical tubes with small holes in lid, lyophilised (24–48 h, Alpha 1-4LD plus) and DNA extracted using DNeasy Plant Mini Kit (Qiagen, Hilden, Germany) according to manufacturers’ instructions. Transformed individuals were identified by polymerase chain reaction (PCR) for the hygromycin gene (*hph*; fwd 5′-tgtcgtccatcacagtttgc-3′, rev 5′-gcgccgatggtttctacaaa-3′) and/or the candidate *jtmD* gene fragments (*jtmD* (129bp); fwd 5′-cacacagcccaagattgcat-3′, rev 5′-tggaagtctatcgccactgg-3′; *jtmD* (432bp); fwd 5′-ggagttcagtgcatgctcag-3′, rev 5′-ggcaagaagaaaggctcacc-3′) carried by the RNA silencing vectors. PCR components and cycling conditions using the CFX Connect™ Real-Time PCR detection system (Bio-Rad, Hercules, CA, USA): 2× FastStart SYBR Green master mix (Roche, Basel, Switzerland), 10 µM forward and reverse primers, 2 μL template DNA, sterile ddH_2_0 (V_T_ 10; 95°C 10 min, (95 °C 30 s, 60 °C 60 s, 72 °C 30 s) × 40, melt curve 60–95 °C (0.5 °C increments) 5 min. Four transgenic endophyte strains were selected for inoculation into perennial ryegrass cv. Alto; E1^jtmD 129bp^, E1^jtmD 432bp-1^, E1^jtmD 432bp-2^, E1^jtmD 432bp-3^.

### 2.4. Generation and Analysis of Perennial Ryegrass Infected with *jtmD* Knockdown Strains

#### 2.4.1. Seedling Inoculation of Transgenic Endophytes

Ryegrass seedlings (30–103) were inoculated with each transgenic endophyte strain, together with the non-transgenic counterpart [49]. Three tiller samples (c. 0.5 cm from the base) of each endophyte-inoculated (25 Alto-E1^jtmD 129bp^, 25 Alto-E1^jtmD 432bp-1^, 27 Alto-E1^jtmD 432bp-2^, 30 Alto-E1^jtmD 432bp-3^ and 10 Alto-E1) and endophyte-free plant (5 Alto-WE) were harvested, following three months growth in soil. DNA extraction [7] and PCR analysis for the transgenes (described above) were performed. All E^+^ plants (plants PCR positive for the transgenes *hph* and *jtmD*, as described previously), from each host-endophyte-combination and Alto-WE were transplanted into 8 cm × 8 cm pots and maintained under glasshouse conditions [49].

#### 2.4.2. Sample Preparation for Epoxy-Janthitrem Profiling

Six-month-old plants (c. 0.5 cm from the base) were lyophilised (48 h, Alpha 1-4LD plus) and ground to a fine powder (1500 rpm for 2 min) (Genogrinder 2010; Spex SamplePrep, Metuchen, NJ, USA). Ground plants (20 mg ± 0.2 mg) were extracted twice with 1 mL 80% methanol (methanol:water, 80:20, *v*:*v*) by vortexing, sonicating and centrifuging for 5 min each. The supernatants were dried (SpeedVac Concentrator, Savant SPD 2010; Thermo Fisher Scientific, Walthman, MA, USA) at 21 °C for 16 h. Extracts were re-constituted in 200 µL of 80% methanol and transferred to liquid chromatography-mass spectrometry (LC-MS) vials.

#### 2.4.3. LC-MS Analysis

Extracts were analysed using a 100 mm × 2.1 mm Hypersil Gold 1.9 µm HPLC column fitted to a Scientific Vanquish liquid chromatograph (Thermo Fisher Scientific, Walthman, MA, USA). The mobile phase was: A (0.1% formic acid in water) (Thermo Fisher Scientific, Walthman, MA, USA), and B (0.1% formic acid in acetonitrile) (Thermo Fisher Scientific, Walthman, MA, USA), at 0.3 mL/min. Initial conditions were 98% A, before initiating a linear gradient to 0% A over 11 min, and this was maintained for 4 min before returning to the initial gradient conditions. Extracts (3 µL) were injected onto the LC-MS system and analysed using a QExactive Plus mass spectrometer (Thermo Fisher Scientific, Walthman, MA, USA) in positive electrospray ionisation (ESI+) mode over a mass range of 80–1200 *m/z*. The MS parameters were as follows: Resolution, 35,000; normalised collision energy, 30 V and the maximum ion time, 200 ms. The source heater temperature was maintained at 310 °C and the heated capillary was maintained at 320°C. The sheath, auxiliary and sweep gases (N_2_) were 28, 15 and four units, respectively. Spray voltage was set at 3.6 kV. Data-dependent MS/MS (MS^2^) spectra were acquired in a separate analysis on a selected Alto-E1 control sample, with compounds of interested targeted. The resolution was set at 17,500 with a normalised collision energy of 30 V, maximum ion time of 50 µs and an isolation window of 1 *m/z*. All LC-MS data were acquired and inspected in Xcalibur Qual Browser v.3.0.63 (Thermo Fisher Scientific, Walthman, MA, USA). The metabolites were defined as MS1 or MS2 level metabolites, according to [51]; the elution time for paxilline was compared with a commercially available standard (HPLC grade ≥98% pure) (Sigma-Aldrich, St. Louis, MO, USA), whilst epoxy-janthitrems I–IV were determined by accurate mass (Δ 0.3–5 ppm) and LC-MS/MS fragmentation patterns matched to the literature [11]. Metabolite abundance (expressed as peak area) was determined for paxilline and epoxy-janthitrems I–IV using Xcalibur LCquan v. 2.7.020 (Thermo Fisher Scientific, Walthman, MA, USA). Statistical differences between the control plants (Alto-E1) and the *jtmD* hairpins were determined at *p* < 0.01 (Student’s *t*-test, unpaired, two tailed distribution).

## 3. Results and Discussion

Since the identification of the biosynthetic pathway for paxilline in *P. paxilli* [29,30,31,52], gene functionality in other indole diterpene biosynthetic pathways, including lolitrem B in *Epichloë* spp. [38], shearinine K and penitrem in *Penicillium* spp. [33,53], aflatrem in *A. flavus* [32] and more recently nodulisporic acid in *Hypoxylon pulicicidum* [54] have been elucidated. These pathways share features in common; orthologous genes that encode enzymes for indole diterpene biosynthesis and genes located in clusters in fungal genomes. More recently, with the availability of whole genome sequence for an increasing number of fungal species, secondary metabolite gene clusters have been observed to be enriched in telomere-proximal regions [55]. In this study, we used this knowledge to identify the JTM locus containing the gene set required for epoxy-janthitrem biosynthesis in *Epichloë*.

### 3.1. Identification of a Candidate Gene, *jtmD*, for Epoxy-Janthitrem Biosynthesis

Qualitative variation for the capacity to produce a specific metabolite appears to be largely attributable to the endophyte genotype, and presumably reflects the presence or absence of the relevant biosynthetic genes. Indole diterpenes, epoxy-janthitrems and lolitrem B share common biosynthetic precursors. Genes from the lolitrem B pathway were used to identify candidates for epoxy-janthitrem biosynthesis.

*Lp*TG-3 and *Lp*TG-4 endophytes do not produce lolitrem B in association with perennial ryegrass. However, these taxa contain all the *ltm* genes except LTM3 cluster genes, *ltmE* and *ltmJ* [7,10,56]. Young et al. [57], also observed that *Lp*TG-3 strain AR37 was missing the genes, *ltmE* and *ltmJ*. Genome sequence analysis was used to identify candidate genes for epoxy-janthitrem biosynthesis in the NEA12 genome. The protein sequences of LtmE and LtmJ from Standard Endophyte (SE), a *Lp*TG-1 strain with similarity to Lp19 [35], were used as query sequences to search the predicted protein database derived from the NEA12 genome. Using this approach, BLASTp searches yielded putative LtmE and LtmJ protein homologues in the library of predicted NEA12 proteins. A search for candidates for epoxy-janthitrem I production unique to *Lp*TG-3 and *Lp*TG-4 genomes yielded a single LtmE homologue and, therefore, the best likely candidate for further investigation.

**Figure 2:**
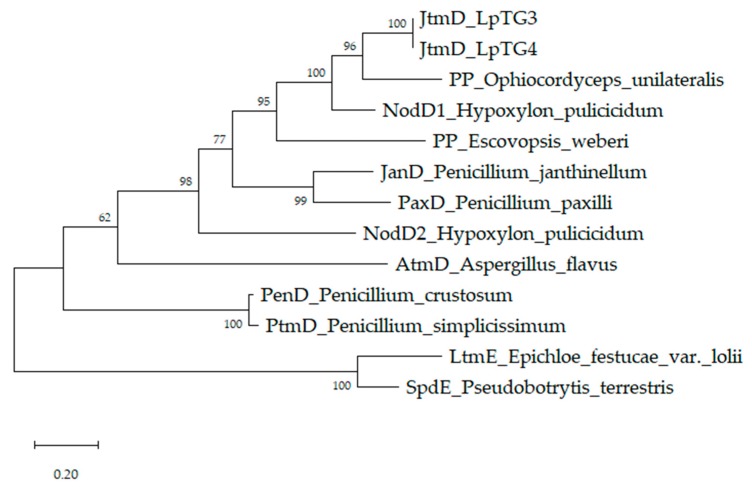
JtmD exhibits amino acid sequence similarity to aromatic prenyl transferases. Tree generated through maximum likelihood (ML) analysis of the predicted 420 amino acid sequence of JtmD from *Lp*TG-3 (NEA12) and *Lp*TG-4 (E1) and selected aromatic prenyl transfereases from filamentous fungi. Fungal species, Genbank accession number and percent amino acid identity to JtmD created by Clustal2.1 is provided for each protein: PP (*O. unilateralis;* PFH61587.1; 67%); NodD1 (*H. pulicicidum;* AUM60056.1; 67%); NodD2 (*H. pulicicidum;* AUM60055.1; 44%); JanD (*P. janthinellum;* AGZ20478.1; 49%); PaxD (*P. paxilli;* Q9C451.2; 46%); PP (*E. weberi;* KOS22745.1; 51%); PtmD (*P. simplicissimum;* BAU61555.1; 31%); PenD (*P. crustosum;* AGZ20194.1; 30%); AtmD (*A. flavus;* A9JPE1.2; 30%); LtmE (*E. festucae var. lolii;* ABF20220.1; 23%); SpdE (*P. terrestris* (BBD84645.1; 23%). The predicted protein sequence of JtmD from NEA12 (MN508661) and E1 (MN508662) are identical. PP: Predicted protein.

The predicted protein sequence of the candidate gene has homology to aromatic prenyl transferases from *H. pulicicidum* (NodD1; amino acid identity 67.23%), *P. janthinellum* (JanD; 49.39%) and *P. paxilli* (PaxD; 46.19%) [33,54] (Figure 2). The genes, *nodD1*, *janD* and *paxD*, are associated with synthesis of the indole diterpenes; nodulisporic acid F, shearinine K and paxilline, respectively. Following this nomenclature, the candidate gene was named *jtmD*.

### 3.2. Identification of the JTM Locus in the *Lp*TG-3 and *Lp*TG-4 Genomes

The NEA12 and E1 genomes were sequenced using long read sequence technology and the contig containing the putative epoxy-janthitrem biosynthetic gene cluster (JTM locus) was identified using the *jtmD* gene sequence as a query. The gene content of the NEA12 and E1 contigs containing *jtmD* was then annotated using a combination of both Augustus gene prediction and manual annotation using the known gene sequences of *ltm* genes and *jtmD* [35,38,45] (Table 2, Figure S1).

The JTM locus is located telomere proximal (6,230,165–6,396,207 bp) in the 6,405,645 bp contig 3 of E1. The homologous chromosome in the *Epichloë* (Fl1) reference genome is the 6 Mb chromosome III [55,58], which harbors the LTM locus. As previously reported for the LTM locus, the JTM locus is characterized by retrotransposon-rich and AT-rich regions [35,38]. The topology of the partial LTM locus in *Lp*TG-3 and *Lp*TG-4 exhibits more similarity to the *E. festucae* LTM locus than the *Lp*TG-1 LTM locus which has two retrotransposon relics inserted between *ltmK* and the *pks* pseudogene. Given the similarity between the *Lp*TG-3 and *Lp*TG-4 loci, it is likely that in addition to these two asexual taxa there is, or once was, a common ancestral sexual *Epichloë* species that synthesized epoxy-janthitrems.

**Figure 3:**
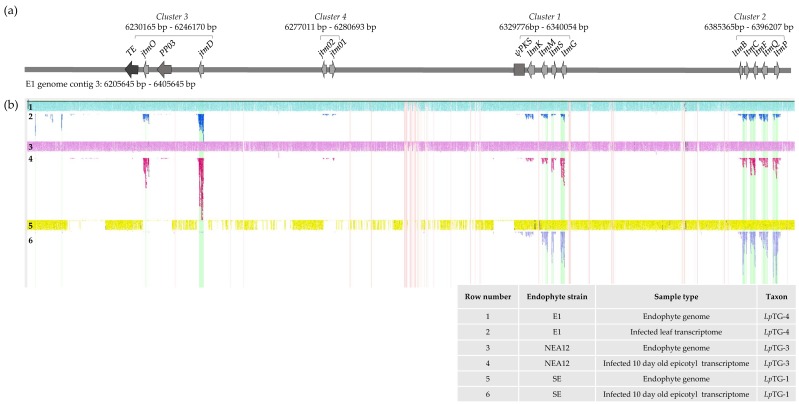
(**a**) Physical map of the JTM locus in the E1 genome. The JTM locus has 13 predicted and known genes in four clusters. Light grey arrows display genes and their orientation. PP = predicted protein; TE = transposable element; ψ = pseudogene. (**b**) In planta expression of genes located at the JTM locus. Gydle (Gydle Inc., Québec City, QC, Canada) output showing RNA-seq reads mapped to the JTM locus (refer to key). Genes located in the JTM locus of E1 and NEA12 are expressed in planta, and absent SE.

The JTM locus contains 13 predicted and known genes in four clusters (Figure 3a, Figure S1). The order and orientation of genes within Cluster 1 (*ltmG, ltmS, ltmM, ltmK*) and Cluster 2 (*ltmP, ltmQ, ltmF, ltmC, ltmB*) are maintained as compared to the *Lp*TG-1 (Lp19, SE) and *E. festucae* (Fl1) LTM loci [35,59]. The 6 genes, *ltmG*, *ltmC, ltmM, ltmB, ltmP* and *ltmQ*, required for paxilline biosynthesis in *Epichloë* spp. are highly conserved with 99%–100% amino acid identity to genes in the LTM locus of *Lp*TG-1 (Table 2). Hence, use of *ltm* nomenclature has been retained in this study.

The *pks* pseudogene defines the boundary between sequence in common to *Lp*TG-1, *Lp*TG-2, *Lp*TG-3 and *Lp*TG-4 genomes and a previously undescribed genome sequence unique to epoxy-janthitrem producing strains (Figure 3a). This region is characterized by four genes, a transposase with a MULE domain, a Helitron helicase-like transposable element, and AT-rich regions. Two gene clusters, termed Cluster 3 and Cluster 4, were identified; none of the genes in this region have been previously described in *Epichloë* endophytes.

Cluster 3 contains two genes, *jtmD* and *jtmO*. As described above, JtmD exhibits highest homology to aromatic prenyl transferases (Figure 2). JtmO exhibits highest homology to NodO from *H. pulicicidum* (69.8%) (Table 2; Figure S2A). JtmO also has homology to JanO**, a FAD-binding oxidoreductase associated with synthesis of shearinines in *P. janthinellum* (52%) [33]. Genes with similar predicted functions have been identified in other indole diterpene gene clusters. For example, the paxilline biosynthetic gene cluster of *P. paxilli* (*paxO*, previously referred to as *PP121*) [29] and the penitrem biosynthetic gene cluster of *P. crustosum* (*penO*) [33]. Similarly, to these proteins, the JtmO protein product is likely to have a role in modification of the indole diterpene core.

Homologues of JtmD and JtmO in *Penicillium* species are often found located side by side. It is interesting to note that in the *E. weberi* genome (GenBank: LGSR01000002.1) [60], the two gene homologues identified in this study (JtmD, predicted protein KOS22745.1; JtmO, predicted protein KOS22754.1) are also found adjacent to each other. Despite having a reduced genome size and gene content in comparison to less specialized relatives, *E. weberi* retains genes necessary for production of toxins, including indole diterpenes such as shearinines [61,62].

The high identity of JtmD to NodD1, and JtmO to NodO [54], is interesting. Nodulisporic acids are a group of emindole SB-like indole diterpenes that are only produced by a monophyletic lineage of asexual endophytic fungal strains widely distributed in the tropics, *H. pulicicidum* [54,63]. Nodulisporic acids are of significance because of their highly potent insecticidal activity [63,64].

Cluster 4 contains two predicted genes, *jtm01* and *jtm02*. The Jtm01 protein shows homology to predicted cytochrome P450 proteins in fungi (Table 2; Figure S2B). Jtm01 does not have an ortholog in any other indole diterpene gene cluster characterized to date. There are, however, predicted cytochrome P450 monooxygenases within other indole diterpene gene clusters that appear to be unique to each cluster, for example PJ-13 within the *P. janthinellum* JAN locus [33], and AF115 in the ATM1 locus [32]. The role of Jtm01 is not clear based on sequence homology, however, as the predicted protein shows some similarity to cytochrome P450 6A1 proteins in other fungi (e.g., *Ophiocordyceps sinensis* CO18, accession EQL02233; Figure S2B), it may have a role in epoxidation [65].

The Jtm02 protein does not exhibit significant homology to any protein sequences in the GenBank database, however, it does show some similarity to proteins predicted to be membrane bound O-acyl transferase (MBOAT) proteins in other fungi (Figure S2C). While the role of Jtm02 is not clear based on sequence homology, as an acyl transferase Jtm02 is most likely associated with the acetylation of the janthitrems and epoxy-janthitrems.

Thus, the predicted genes for epoxy-janthitrem biosynthesis are located at a single locus, JTM, within the *Lp*TG-3 and *Lp*TG-4 genomes, as is the case for previously characterized indole diterpenes including lolitrem B [35] and paxilline [31].

### 3.3. Transcriptome Expression of Genes Located in the Janthitrem Biosynthesis Gene Cluster

Following identification of four gene clusters using whole genome sequence analysis, transcriptome analysis of candidate genes for epoxy-janthitrem biosynthesis was performed to study their expression in planta. This was achieved by mapping RNAseq reads generated from perennial ryegrass-endophyte associations, NEA12, E1 and SE to the E1 JTM locus (Figure 3b). Mapping of reads to previously defined Cluster 1 and Cluster 2 genes was observed for all three ryegrass-endophyte associations. In addition, reads were mapped to the genes proposed to be involved in epoxy-janthitrem biosynthesis, *jtmD*, *jtmO*, *jtm01*, and *jtm02* only in NEA12 and E1 associations, confirming expression in planta. RNAseq reads generated from the perennial ryegrass-SE association were not mapped to Cluster 3 and Cluster 4 genes further confirming the absence of these two clusters.

### 3.4. Functional Analysis of *jtmD* to Determine Involvement in Epoxy-Janthitrem Biosynthesis

**Figure 4:**
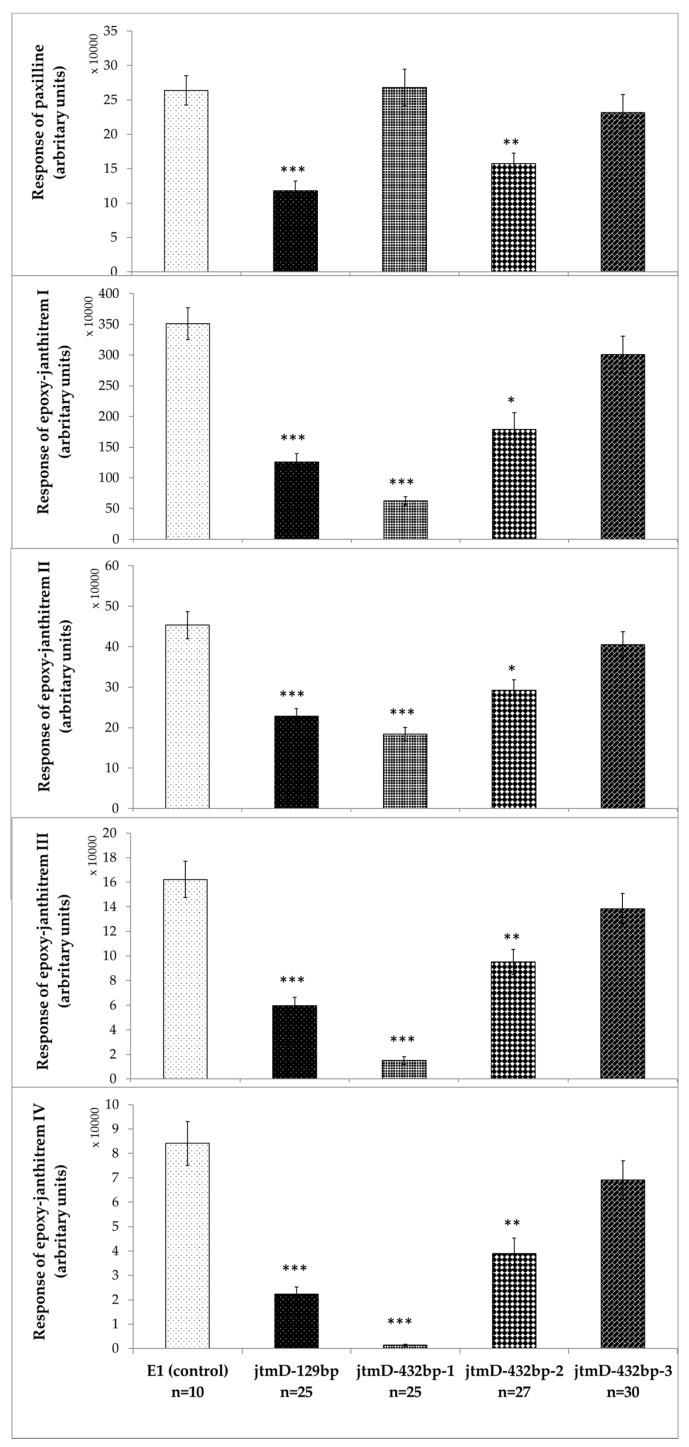
In planta assessment of paxilline and epoxy-janthitrem I–IV production in Alto-E1 host-endophyte associations with *jtmD* hairpins. The response denotes arbitrary units, measured as chromatography peak area (peak height by width). Bars represent standard error of the mean (SEM). Significance is measured by Student’s unpaired *t*-test and directly compares the Alto-E1*^jtmD^* ^RNAi^ hairpins to Alto-E1. * *p* < 0.01; ** *p* < 0.001, *** *p* < 0.0001. *n* = the number of individual perennial ryegrass-endophyte associations measured.

*Lp*TG-4 endophyte strain E1 was modified, using RNAi, to determine the involvement of *jtmD* in epoxy-janthitrem biosynthesis. Following seedling inoculation, in planta production of epoxy-janthitrem I–IV was measured in individually inoculated plants of Alto-E1 (non-transgenic control) and Alto-E1*^jtmD^* ^RNAi^ (E1^jtmD 129bp^, E1^jtmD 432bp-1^, E1^jtmD 432bp-2^ and E1^jtmD 432bp-3^) symbiota (Figure 4). LC-MS/MS fragmentation patterns, as defined by Tapper et al. [11], were used to assign the metabolites (Figure S3,). Epoxy-janthitrem I, comprising 83.5% of the total epoxy-janthitrems I–IV, was the most abundant epoxy-janthitrem in Alto-E1 symbiota. The efficacy of the RNAi knockdown, as measured by the abundance of epoxy-janthitrems I–IV, was assessed between the two constructs (E1^jtmD 129bp^ and E1^jtmD 432bp^) as well as between independent fungal transformants of the same construct (E1^jtmD 432bp-1^, E1^jtmD 432bp-2^ and E1^jtmD 432bp-3^). Variation in RNAi knockdown efficiency was observed, as would be expected; for example, the E1^jtmD 432bp^ RNAi construct was effective in downregulating *jtmD*, as measured by epoxy-janthitrem abundance, in transformants 1 and 2, but not 3.

The abundance of epoxy-janthitrem I in Alto-E1^[*jtmD* RNAi]^ symbiota was reduced when compared to non-transgenic E1 control plants, with significant differences observed for Alto-E1^jtmD 129bp^ (*p* < 0.0001; 64% reduction), Alto-E1^jtmD 432bp-1^ (*p* < 0.0001; 82% reduction) and Alto-E1^jtmD 432bp-2^ (*p* < 0.01; 49% reduction) symbiota (Figure 4). The same pattern of RNAi efficacy in reducing epoxy-janthitrem abundance was observed for epoxy-janthitrems II–IV. The significant reduction in epoxy-janthitrems I–IV indicates successful silencing of the *jtmD* gene and implicates JtmD in the biosynthesis of epoxy-janthitrems in *Epichloë* species.

Production of the precursory metabolite paxilline was also measured. Paxilline abundance was perturbed in response to *jtmD* knockdown, however, a significant change in paxilline abundance was only observed when moderate (49%, 64%) reduction of epoxy-janthitrem was observed. When compared to Alto-E1 symbiota, abundance of paxilline was significantly reduced in Alto-E1*^jtmD^* ^129bp^ (*p* < 0.0001; 55% reduction) and Alto-E1^jtmD 432bp-2^ (*p* < 0.001; 40% reduction) symbiota. Interestingly, when compared to the control symbiota, Alto-E1*^jtmD^* ^432bp-1^ symbiota produced the lowest levels of epoxy-janthitrem I–IV (*p* < 0.0001; 82% reduction), yet no change in paxilline abundance was observed in these plants. Effects on upstream components of indole diterpene pathways as result of gene knockdown have not frequently been reported. One such study describes that the disruption of the *ter*P cluster involved in terpendole E biosynthesis resulted in overproduction of upstream alkaloids [36]. The *ter*P gene is involved in the metabolic conversion of terpendole E to its downstream counterparts; thus, *terP* knockout strains of *Agrobacterium tumefaciens* resulted in terpendole E accumulation [36].

### 3.5. Proposed Pathway for Epoxy-Janthitrem Biosynthesis

Indole diterpene gene clusters identified to date are characterized by a core set of four genes for the synthesis of paspaline, and a suite of additional genes that encode multi-functional cytochrome P450 monooxygenases, FAD dependent monooxygenases and prenyl transferases that catalyse various regio- and stereo- specific oxidations on the molecular skeleton to generate a diversity of indole diterpene products [33].

**Figure 5:**
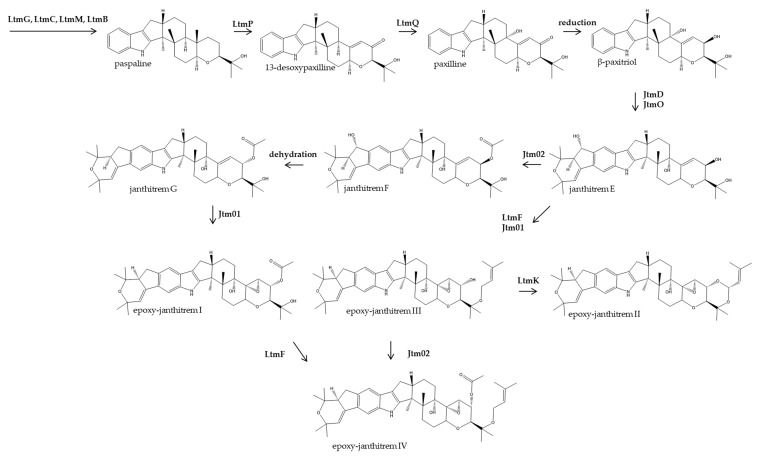
Proposed pathway for epoxy-janthitrem I–IV biosynthesis. The suggested scheme follows the KEGG indole diterpene biosynthetic pathway, illustrating a parsimonious route to epoxy-janthitrem I (11, 12-epoxy-janthitrem G) and its variants (epoxy-janthitrems II–IV). All epoxy-janthitrems were tentatively identified by LCMS/MS and fragment ions were matched [11,67].

A framework for the biosynthesis of the epoxy-janthitrems in *Epichloë* is described based on analogy with other indole diterpene pathways, identification of biosynthesis intermediates using LC-MS, and the functional analysis of *jtmD*, which implicated the gene in epoxy-janthitrem biosynthesis (Figure 5).

Robust high-resolution LC-MS was used to target key metabolites proposed to be associated with the biosynthesis of indole diterpene alkaloids, specifically epoxy-janthitrems (I–IV). The extracted ion chromatograms of the compounds, observed in planta in Alto-E1 symbiota, are illustrated in (Figure S3). The observed retention times were identified by accurate mass (Δ 0.3–5 ppm) and fragmentation patterns (Table 3).

Saikia et al. [30] showed that four genes, *paxG*, *paxM*, *paxB* and *paxC*, are required for the biosynthesis of the first stable indole diterpene intermediate, paspaline. Genetic analysis of *P. paxilli* has established that paxilline biosynthesis then requires two cytochrome p450 monooxygenases, *paxP* and *paxQ* which utilise paspaline and 13-desoxypaxilline as their respective substrates [30]. Orthologues of these six genes are found in all paspaline-derived indole diterpene gene cluster loci characterized to date. In *P. janczewskii* and *P. janthinellum*, incorporation of labelled β-paxitriol, and not α-paxitriol, into penitrem A and E and janthitrem B and C, respectively, suggests that β-paxitriol is an immediate precursor for the complex indole diterpenes with B-stereochemistry [23].

Genetic evidence suggests that the biosynthesis of epoxy-janthitrems proceeds along the same pathway as lolitrem B in other *Epichloë* species, involving *ltmG*, *ltmC*, *ltmM* and *ltmB* in the synthesis of paspaline. Here, we propose that initial steps in the epoxy-janthitrem pathway require LtmP and LtmQ for the demethylation and hydroxylation of paspaline for the synthesis of β-paxitriol. JtmD and JtmO are required for the diprenylation and oxidative cyclisation to construct the distinct A/B ring in the janthitremane indole diterpenes. LtmF and LtmK are required for the prenylation and cyclisation of the epoxy-janthitrems II to IV consistent with the lolitrem biosynthesis (Figure 5). Jtm01 (P450 monoxygenase) is required for epoxidation in the pathway, which is consistent with previous reports [65]. Jtm02 (membrane bound O-acyl transferase), is most likely associated with the acetylation of the janthitrems and epoxy-janthitrems.

## 4. Concluding Remarks

The work described here provides a genetic basis for epoxy-janthitrem biosynthesis in *Epichloë* endophytes. Genes for epoxy-janthitrem biosynthesis are found clustered at a single locus, JTM, on chromosome III of the genome. Functional analysis of the *jtmD* gene using RNAi knockdown, revealed reduced abundance of epoxy-janthitrems I–IV, implicating JtmD in epoxy-janthitrem biosynthesis. While the role of JtmD and JtmO can be predicted based on comparison to previously well characterized indole diterpene pathways, further work is required to determine a role of Jtm01 and Jtm02 in epoxy-janthitrem biosynthesis. A biosynthetic pathway based on predicted protein function and comparison with other indole diterpene pathways is proposed. As the genes for epoxy-janthitrem production are now determined, it becomes possible to predict janthitrem production by screening strains for the presence of *jtm* genes and manipulate the pathway, using genome editing technology. A limitation to the study of epoxy-janthitrems in the past has been their unstable nature once isolated; it is now possible to synthesize sufficient quantities for isolation and use in accurate measurement as well as in insect/animal toxicity studies. There is also potential for creating novel indole diterpenes in *Epichloë* endophytes using the existing LTM gene clusters and adding new designer gene clusters using a targeted genome editing approach. Deeper understanding of epoxy-janthitrem production enables exploiting their potential use in pasture pest management, enhancing pasture persistence while minimizing the threat posed by their likely tremorgenicity.

## Figures and Tables

**Table 1:** Properties of *Epichloë* endophytes.

Taxon ^a^	*E. festucae* var. *lolii* (*Lp*TG-1)	*Lp*TG-3	*Lp*TG-4
Endophyte strain	**SE**	**NEA12**	**E1**
Alkaloid biosynthesis profile ^b^	lolitrem B, ergovaline, peramine	epoxy-janthitrems	epoxy-janthitrems

^a,b^ [6,7]; All isolates provided by Agriculture Victoria Research.

**Table 2:** Sequence analysis of the JTM locus and other features.

Position in JTM Cluster		Top BLASTp Hit					
Feature ID	Gene Cluster	Predicted Function	Homologous Gene	Percent Identity (aa)	Organism	Genbank Accession No.	Reference
*TE*	-	Transposable element		85%	*Hirsutella minnesotensis*	KJZ70955	[66]
*jtmO*	3	FAD-dependent oxygenase	*nodO*	70%	*Hypoxylon pulicicidum*	AUM60052.1	[54]
*PP03*	3	Transposase	hypothetical protein	86%	*Hirsutella minnesotensis*	KJZ68513	[66]
*jtmD*	3	Aromatic prenyl transferase	*nodD1*	67%	*Hypoxylon pulicicidum*	AUM60056.1	[54]
*jtm02*	4	Membrane bound O-acyl transferase	hypothetical protein	34%	*Oidiodendron maius Zn*	KIM95229	unpublished
*jtm01*	4	Cytochrome P450 monooxygenase	hypothetical protein	68%	*Hirsutella minnesotensis*	KJZ77225	[66]
ψ*pks*	-	Polyketide synthase (pseudogene)		73%	*Fusarium equiseti*	ALQ32965.1	unpublished
*ltmK*	1	Cytochrome P450 monooxygenase	*ltmK*	99%	*Lp*TG-1	AY742903	[38]
*ltmM*	1	FAD-dependent monooxygenase	*ltmM*	99%	*Lp*TG-1	AY742903	[38]
*ltmS*	1	Integral membrane protein	*ltmS*	100%	*Lp*TG-1	AY742903	[38]
*ltmG*	1	GGPP synthase	*ltmG*	99%	*Lp*TG-1	AY742903	[38]
*ltmB*	2	Integral membrane protein	*ltmB*	100%	*Lp*TG-1	DQ443465	[35]
*ltmC*	2	Prenyl transferase	*ltmC*	100%	*Lp*TG-1	DQ443465	[35]
*ltmF*	2	Prenyl transferase	*ltmF*	99%	*Lp*TG-1	DQ443465	[35]
*ltmQ*	2	Cytochrome P450 monooxygenase	*ltmQ*	100%	*Lp*TG-1	DQ443465	[35]
*ltmP*	2	Cytochrome P450 monooxygenase	*ltmP*	100%	*Lp*TG-1	DQ443465	[35]

**Table 3:** Proposed metabolites involved in the biosynthesis of the paxilline and epoxy-janthitrem I–IV, assessed in planta using Alto-E1 samples by LC-MS/MS. The accurate masses (*m/z*), retention times (RT) and fragmentation data (product ions, MS^2^) were acquired in positive ionization mode (M+H). Accurate mass and MS^2^ results were compared with theoretical masses and fell within the range of 0.3–5 ppm difference (Δ ppm).

Metabolite	RT (min)	*m/z* (M+H)	Product Ions (MS^2^)				Formula (M+H)	Theoretical Mass (M+H)	Δ (ppm)
			1	2	3	4			
Paxilline *	9.76	436.2481	130.0653	182.0966	288.9844	418.2376	C27 H34 O4 N	436.2482	−0.36
epoxy-janthitrem I	11.07	646.3726	222.1276	280.1694	588.3315	631.3452	C39 H52 O7 N	646.3738	−1.81
epoxy-janthitrem II	12.22	670.4078	222.1277	280.1693	612.3679	655.3818	C42 H56 O6 N	670.4102	−3.63
epoxy-janthitrem III	12.37	672.4230	222.1277	280.1687	614.3801	657.3967	C42 H58 O6 N	672.4259	−4.31
epoxy-janthitrem IV	12.35	714.4329	222.1276	280.1695	656.3939	699.4077	C44 H60 O7 N	714.4364	−3.96

*: matched with known chemical standard.

## Supplementary Materials

The following are available online at https://www.mdpi.com/2076-2607/7/11/560/s1. **Figure S1.** Physical map of the JTM locus in the NEA12 and E1 genomes. The JTM locus has 13 predicted and known genes in four clusters. Cluster 1 (*ltmG*, *ltmS*, *ltmM*, *ltmK*), Cluster 2 (*ltmP*, *ltmQ*, *ltmF*, *ltmC*, *ltmB*), Cluster 3 (*jtmD* and *jtmO*) and Cluster 4 (*jtm01, jtm02*). Light grey arrows display predicted and known genes and their orientation. The locations of the *pks* pseudogene, transposase with a MULE domain (*PP03*) and Helitron helicase-like transposable element (TE) are also shown. PP = predicted protein; TE = transposable element; ψ = pseudogene. **Figure S2.** Trees generated through ML analysis of the predicted amino acid sequence of JTM locus genes from NEA12 and E1 and selected top BLASTp hits in the NCBI database. Genbank accession number and percent amino acid identity created by Clustal2.1, and species name is provided for each protein. (**a**) JtmO, exhibits sequence similarity to FAD-binding oxidoreductases: NodO (AUM60052.1; *H. pulicicidum*; 70%); PP (KOS22754.1; *E. weberi*; 60%); JanO (AGZ20488.1; *P. janthinellum*; 52%); PaxO (ADO29935.1; *P. paxilli*; 49%); PtmO (BAU61564.1; *P. simplicissimum*; 43%); PenO (AGZ20199.1; *P. crustosum*; 43%). The predicted protein sequence of JtmO from NEA12 (MN508663) and E1 (MN508664) are 99.79% identical; Jtm01 from E1 is 470 amino acids, the 479 aa NEA12 protein has a n amino acid insertion at position 12 and an amino acid variation (T/A) at position 326. (**b**) Jtm01, with a 387 aa protein sequence, exhibits sequence similarity to cytochrome P450 monoxygenases: KJZ77225.1 (68%; *H. minnesotensis* 3608); EQL02233.1 (57%; *Ophiocordyceps sinensis* CO18); KND87478.1 (53%; *Tolypocladium ophioglossoides* CBS 100239); OAQ66296.1 (50%; *Pochonia chlamydosporia* 170); KOM22171.1 (55%; *O. unilateralis*); XP_013947710.1 (48%; *Trichoderma atroviride* IMI 206040). The predicted protein sequence of Jtm01 from NEA12 (MN508667) and E1 (MN508668) are identical. (**c**) Jtm02, with a 315 aa protein sequence, exhibits sequence similarity to MBOAT proteins: KIM95229.1 (33%; *Oidiodendron maius* Zn); KZL85868.1(30%; *Colletotrichum incanum*); CCX05903.1 (30%; *Pyronema omphalodes* CBS 100304); KZP09605.1 (29%; *Fibulorhizoctonia* sp. CBS 109695); XP_007593790.1 (31%; *Colletotrichum fioriniae* PJ7). The predicted protein sequence of Jtm02 from NEA12 (MN508665) and E1 (MN508666) are identical. PP: Predicted protein. **Figure S3.** Extracted ion chromatogram (EIC) and MS^2^ fragments of: (**a**) paxilline in planta and matched to a chemical standard *m/z* 436.2483 at 9.76 min; (**b**) epoxy-janthitrem I in planta, *m/z* 646.3726 at 11.07 min; (**c**), epoxy-janthitrem II in planta, *m/z* 670.4078 at 12.22 min; (**d**) epoxy-janthitrem III in planta, *m/z* 672.4221 at 12.37 min; and (**e**) epoxy-janthitrem IV in planta, *m/z* 714.4329 at 12.35 min. Metabolites were observed in perennial ryegrass- *Lp*TG-4 associations (Alto-E1), collected from 0–20 min in positive ionisation mode (ESI+).
