# Supplementary material for: Analysis of the Indole Diterpene Gene Cluster for Biosynthesis of the Epoxy-Janthitrems in Epichloë Endophytes
Source: Microorganisms. 2019 Nov 13;7(11):560. doi: 10.3390/microorganisms7110560 (PMC6921081; doi:10.3390/microorganisms7110560)
Supplement: Supplementary file 1 [file microorganisms-07-00560-s001.pdf]

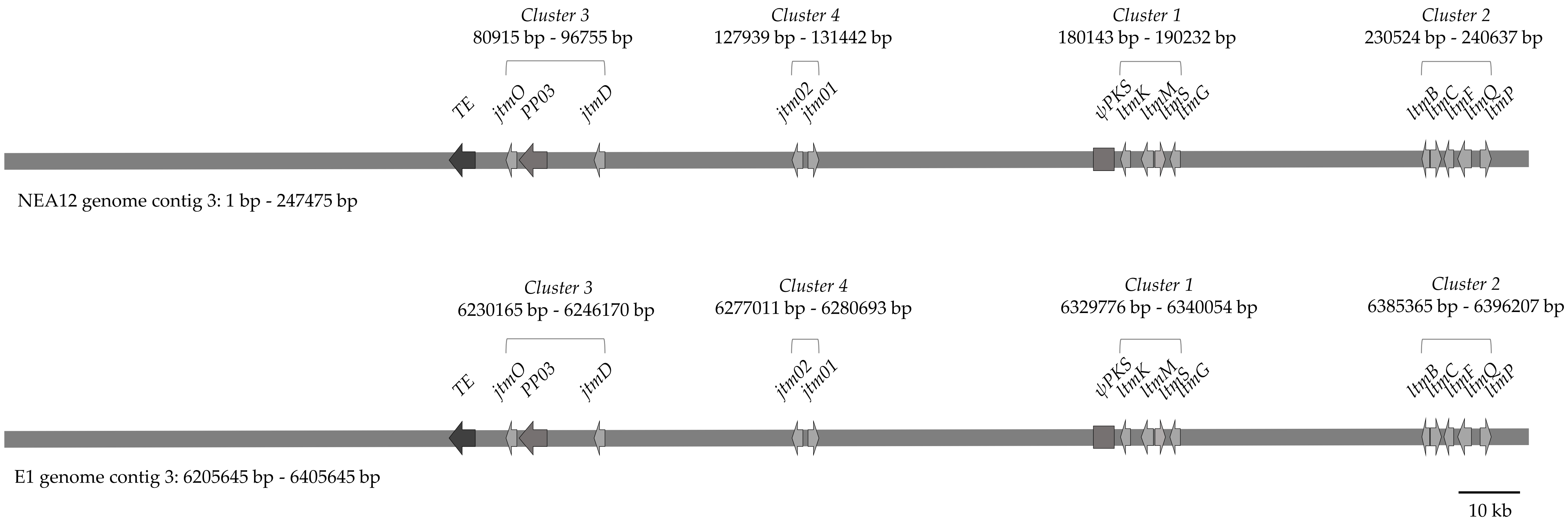

Figure S1. Physical map of the JTM locus in the NEA12 and E1 genomes. The JTM locus has 13 predicted and known genes in four clusters. Cluster 1 (*ltmG*, *ltmS*, *ltmM*, *ltmK*), Cluster 2 (*ltmP*, *ltmQ*, *ltmF*, *ltmC*, *ltmB*), Cluster 3 (*jtmD* and *jtmO*) and Cluster 4 (*jtm01*, *jtm02*). Light grey arrows display predicted and known genes and their orientation. The locations of the *pks* pseudogene, transposase with a MULE domain (*PP03*) and Helitron helicase-like transposable element (TE) are also shown. PP = predicted protein; TE = transposable element; ψ = pseudogene.

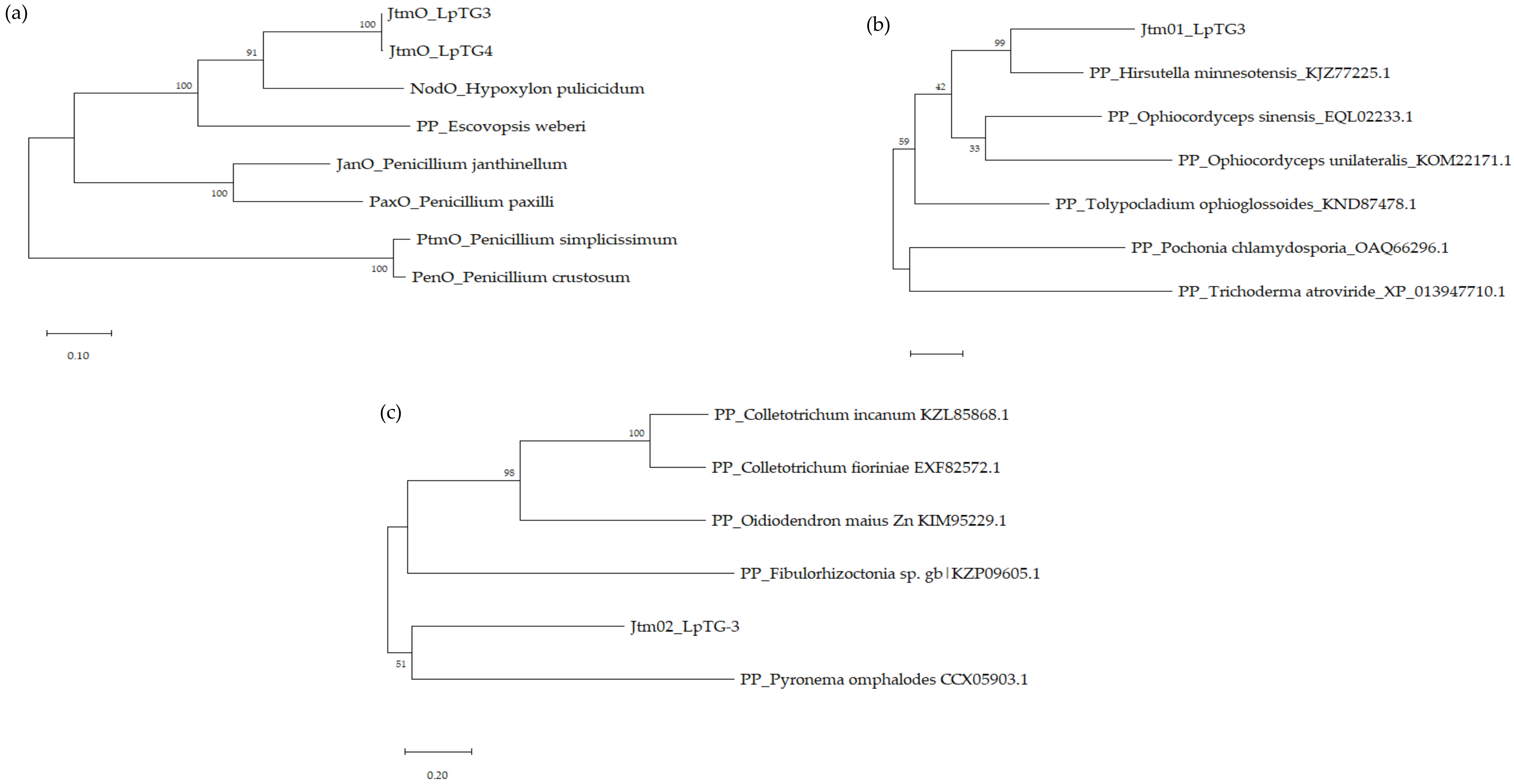
(a)
JtmO_LpTG3
JtmO_LpTG4
NodO_Hypoxylon pulicicidum
PP_Escovopsis weberi
JanO_Penicillium janthinellum
PaxO_Penicillium paxilli
PtmO_Penicillium simplicissimum
PenO_Penicillium crustosum
100
91
100
100
100
0.10
(b)
Jtm01_LpTG3
PP_Hirsutella minnesotensis_KJZ77225.1
PP_Ophiocordyceps sinensis_EQL02233.1
PP_Ophiocordyceps unilateralis_KOM22171.1
PP_Tolypocladium ophioglossoides_KND87478.1
PP_Pochonia chlamydosporia_OAQ66296.1
PP_Trichoderma atroviride_XP_013947710.1
99
42
33
59
(c)
PP_Colletotrichum incanum KZL85868.1
PP_Colletotrichum fioriniae EXF82572.1
PP_Oidiodendron maius Zn KIM95229.1
PP_Fibulorhizoctonia sp. gb|KZP09605.1
Jtm02_LpTG-3
PP_Pyronema omphalodes CCX05903.1
100
98
51
0.20

Figure S2. Trees generated through ML analysis of the predicted amino acid sequence of JTM locus genes from NEA12 and E1 and selected top BLASTp hits in the NCBI database. Genbank accession number and percent amino acid identity created by Clustal2.1, and species name is provided for each protein**.** (**a**) JtmO, exhibits sequence similarity to FAD-binding oxidoreductases: NodO (AUM60052.1; *H. pulicicidum*; 70%); PP (KOS22754.1; *E. weberi*; 60%); JanO (AGZ20488.1; *P. janthinellum*; 52%); PaxO (ADO29935.1; *P. paxilli*; 49%); PtmO (BAU61564.1; *P. simplicissimum*; 43%); PenO (AGZ20199.1; *P. crustosum*; 43%). The predicted protein sequence of JtmO from NEA12 (MN508663) and E1 (MN508664) are 99.79% identical; Jtm01 from E1 is 470 amino acids, the 479 aa NEA12 protein has a n amino acid insertion at position 12 and an amino acid variation (T/A) at position 326. (**b**) Jtm01, with a 387 aa protein sequence, exhibits sequence similarity to cytochrome P450 monoxygenases: KJZ77225.1 (68%; *H. minnesotensis* 3608); EQL02233.1 (57%; *Ophiocordyceps sinensis* CO18); KND87478.1 (53%; *Tolypocladium ophioglossoides* CBS 100239); OAQ66296.1 (50%; *Pochonia chlamydosporia* 170); KOM22171.1 (55%; *O. unilateralis*); XP_013947710.1 (48%; *Trichoderma atroviride* IMI 206040). The predicted protein sequence of Jtm01 from NEA12 (MN508667) and E1 (MN508668) are identical. (**c**) Jtm02, with a 315 aa protein sequence, exhibits sequence similarity to MBOAT proteins: KIM95229.1 (33%; *Oidiodendron maius* Zn); KZL85868.1(30%; *Colletotrichum incanum*); CCX05903.1 (30%; *Pyronema omphalodes* CBS 100304); KZP09605.1 (29%; *Fibulorhizoctonia* sp. CBS 109695); XP_007593790.1 (31%; *Colletotrichum fioriniae* PJ7). The predicted protein sequence of Jtm02 from NEA12 (MN508665) and E1 (MN508666) are identical. PP: Predicted protein.

(a)

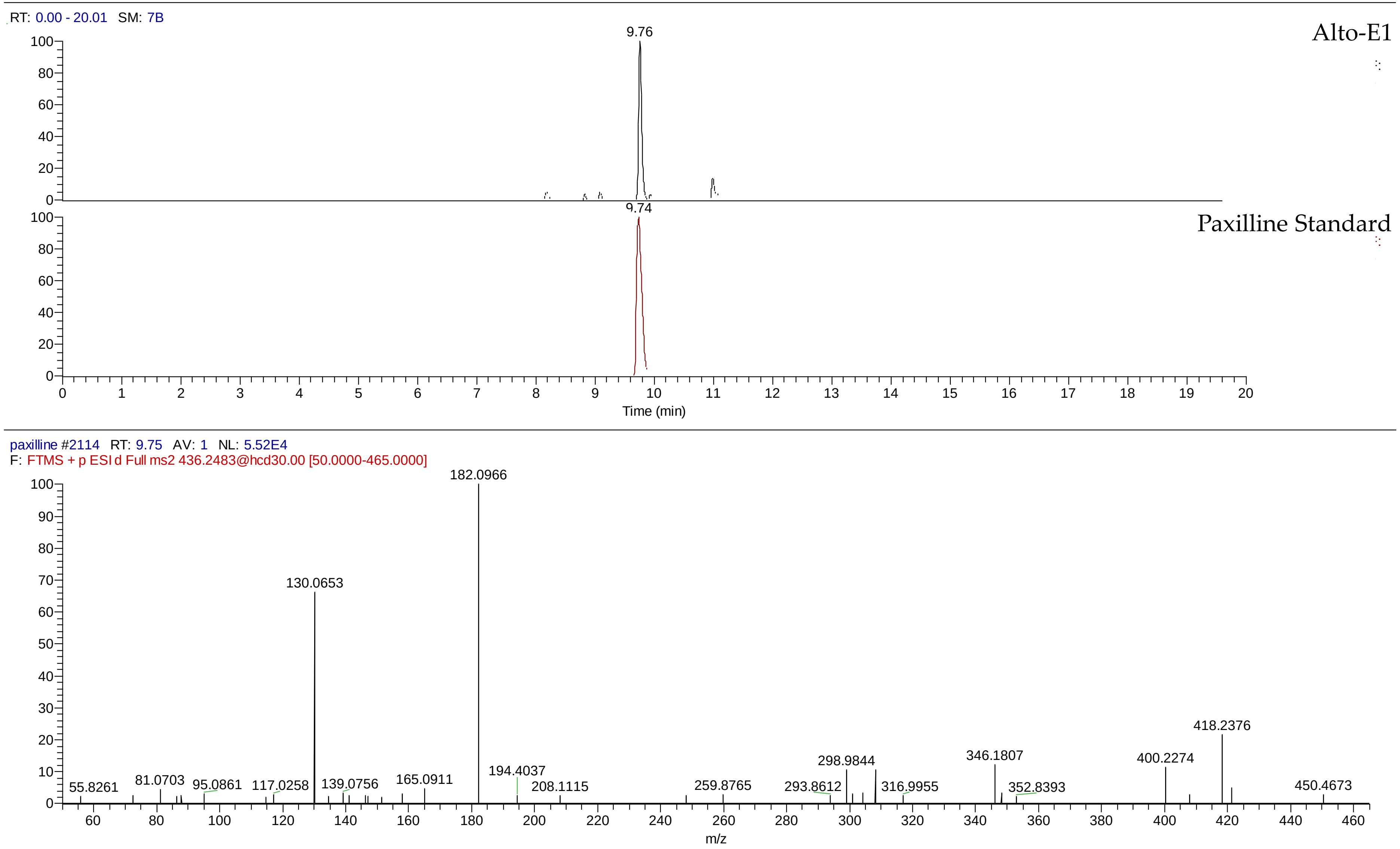
RT: 0.00 - 20.01 SM: 7B
Alto-E1
Paxilline Standard
9.76
9.74
Time (min)
paxilline #2114 RT: 9.75 AV: 1 NL: 5.52E4
F: FTMS + p ESI d Full ms2 436.2483@hcd30.00 [50.0000-465.0000]
182.0966
130.0653
418.2376
346.1807
400.2274
298.9844
194.4037
55.8261
81.0703
95.0861
117.0258
139.0756
165.0911
208.1115
259.8765
293.8612
316.9955
352.8393
450.4673
m/z

(b)

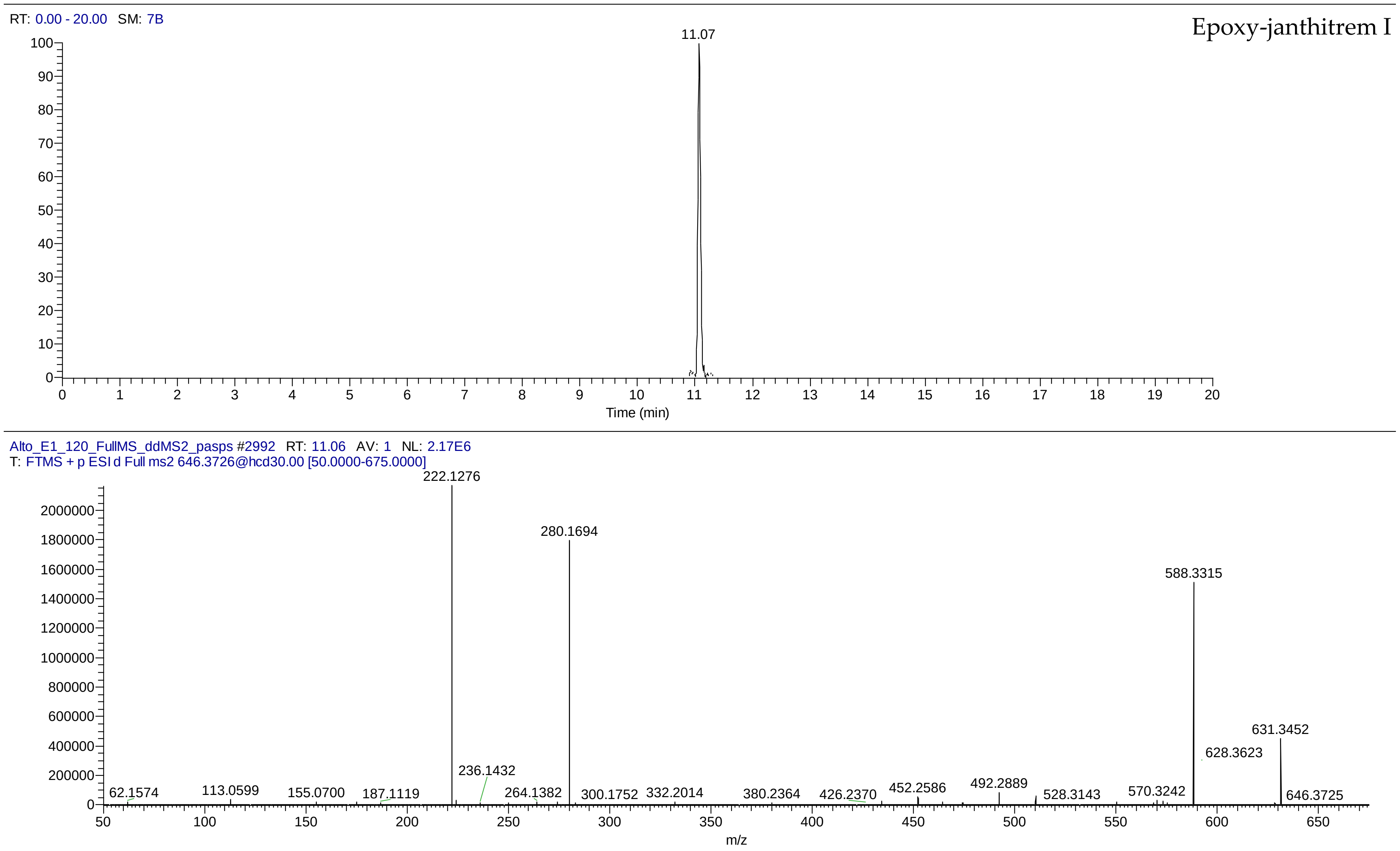

Epoxy-janthitrem I
RT: 0.00 - 20.00 SM: 7B
11.07
Time (min)
Alto_E1_120_FullMS_ddMS2_pasps #2992 RT: 11.06 AV: 1 NL: 2.17E6
T: FTMS + p ESI d Full ms2 646.3726@hcd30.00 [50.0000-675.0000]
222.1276
280.1694
588.3315
631.3452
628.3623
236.1432
62.1574
113.0599
155.0700
187.1119
264.1382
300.1752
332.2014
380.2364
426.2370
452.2586
492.2889
528.3143
570.3242
646.3725
m/z

(c)

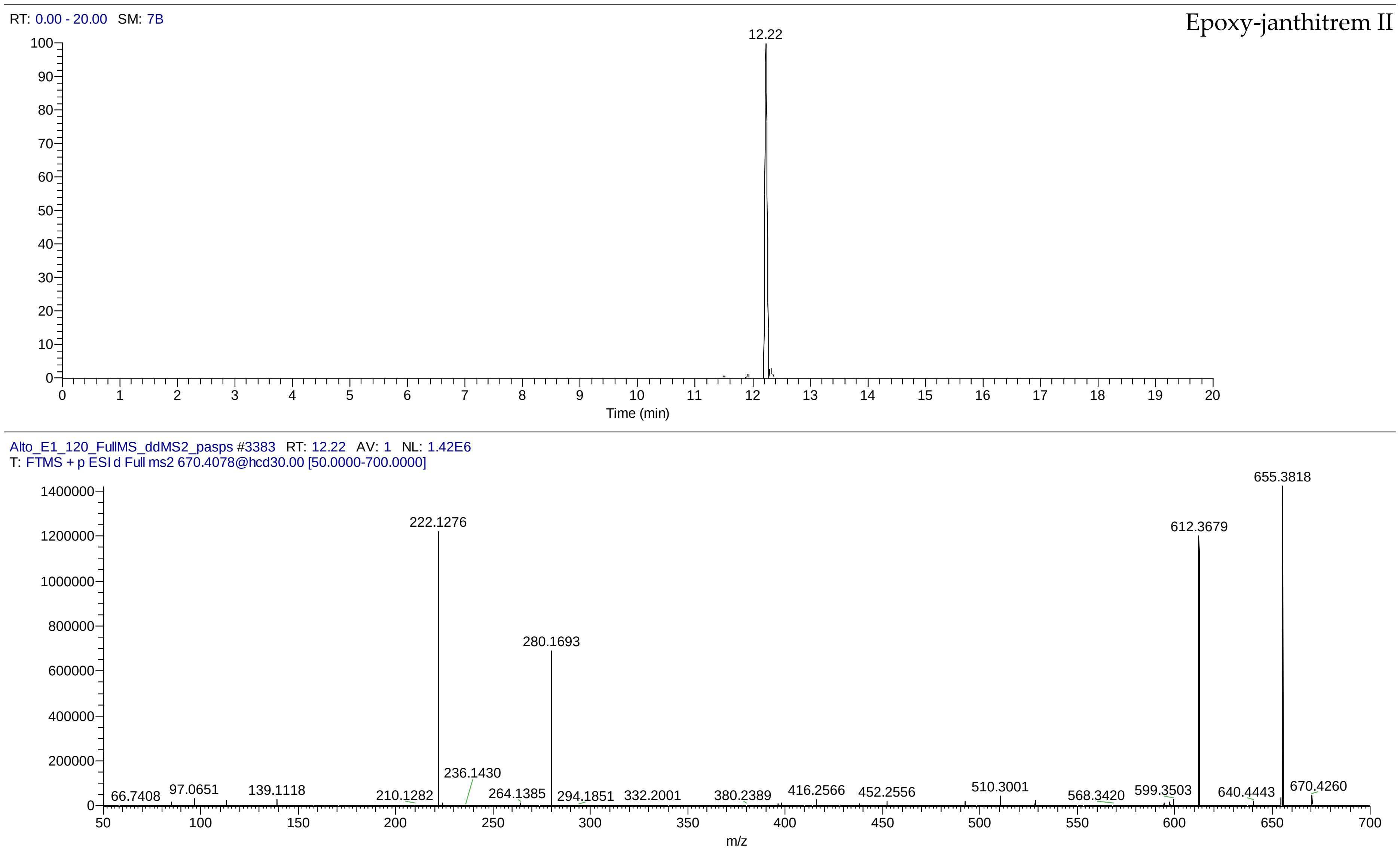

RT: 0.00 - 20.00 SM: 7B
Epoxy-janthitrem II
12.22
Time (min)
Alto_E1_120_FullMS_ddMS2_pasps #3383 RT: 12.22 AV: 1 NL: 1.42E6
T: FTMS + p ESI d Full ms2 670.4078@hcd30.00 [50.0000-700.0000]
655.3818
612.3679
222.1276
280.1693
236.1430
66.7408
97.0651
139.1118
210.1282
264.1385
294.1851
332.2001
380.2389
416.2566
452.2556
510.3001
568.3420
599.3503
640.4443
670.4260
m/z

(d)

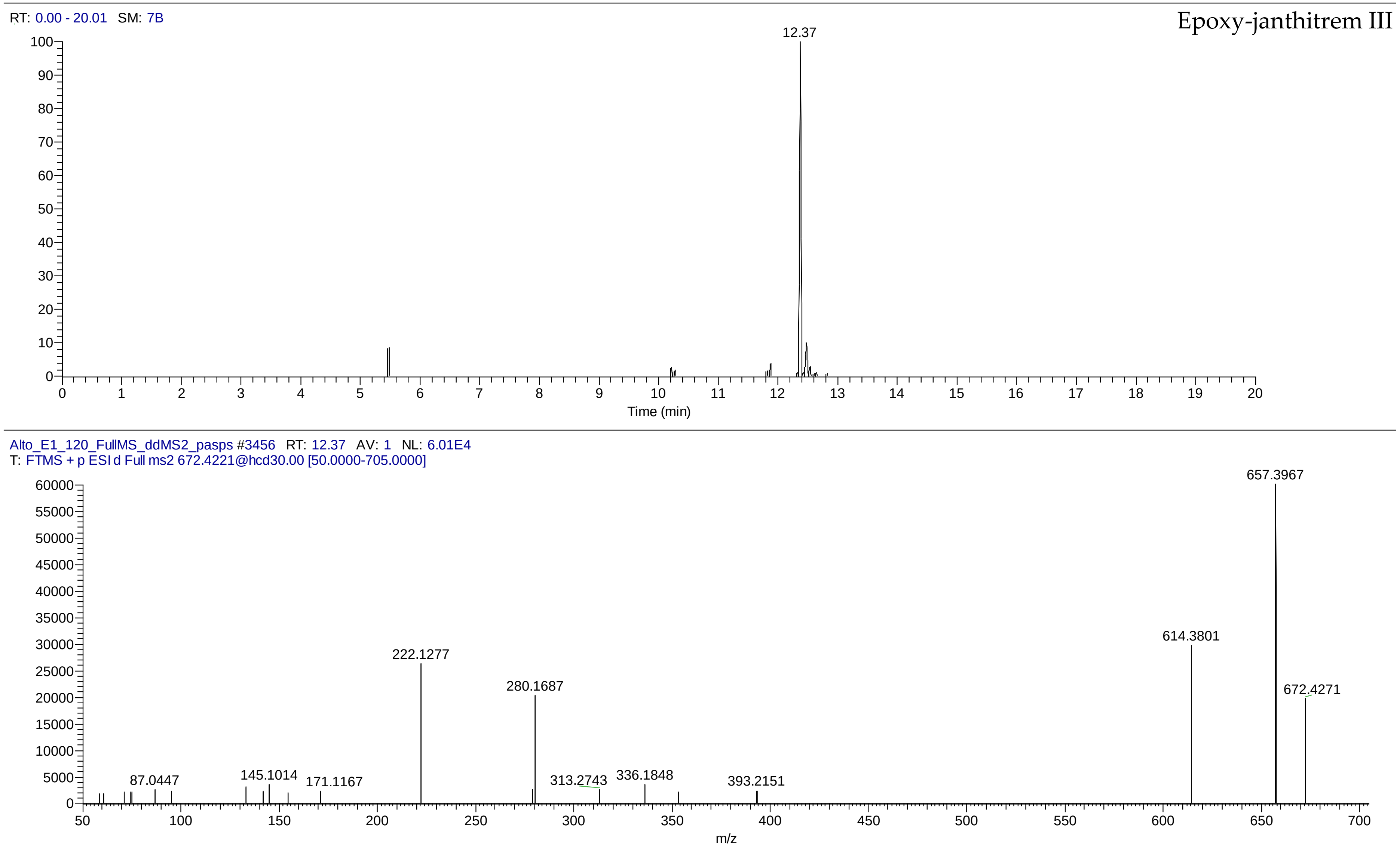

RT: 0.00 - 20.01 SM: 7B
Epoxy-janthitrem III
12.37
Time (min)
Alto_E1_120_FullMS_ddMS2_pasps #3456 RT: 12.37 AV: 1 NL: 6.01E4
T: FTMS + p ESI d Full ms2 672.4221@hcd30.00 [50.0000-705.0000]
657.3967
614.3801
672.4271
222.1277
280.1687
87.0447
145.1014
171.1167
313.2743
336.1848
393.2151
m/z

(e)

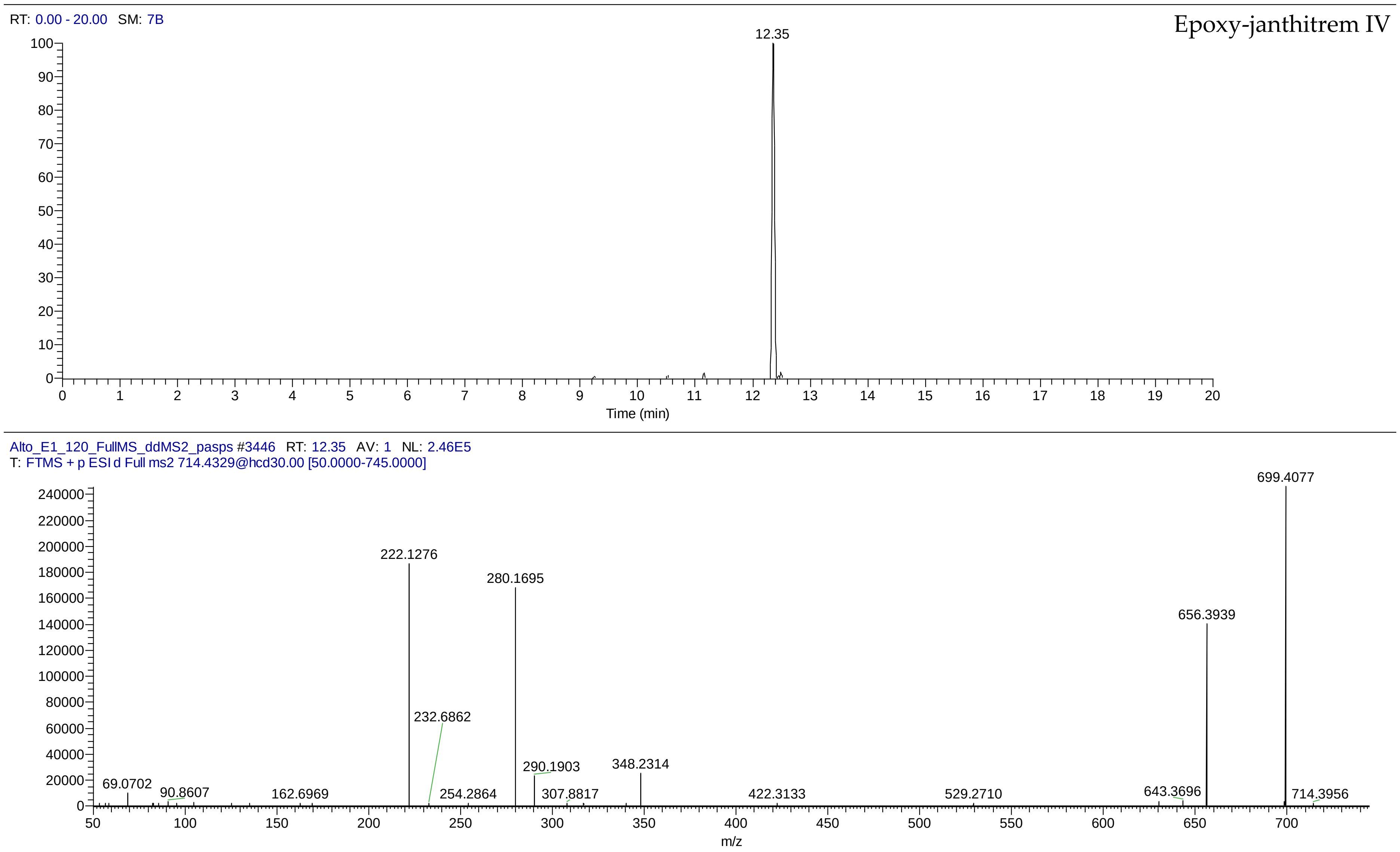

Figure S3. Extracted ion chromatogram (EIC) and MS^2 fragments of: (**a**) paxilline in planta and matched to a chemical standard *m/z* 436.2483 at 9.76 min; (**b**) epoxy-janthitrem I in planta, *m/z* 646.3726 at 11.07 min; (**c**), epoxy-janthitrem II in planta, *m/z* 670.4078 at 12.22 min; (**d**) epoxy-janthitrem III in planta, *m/z* 672.4221 at 12.37 min; and (**e**) epoxy-janthitrem IV in planta, *m/z* 714.4329 at 12.35 min. Metabolites were observed in perennial ryegrass- *Lp*TG-4 associations (Alto-E1), collected from 0–20 min in positive ionisation mode (ESI+).
